# Recent advances in diagnostic technologies for postoperative central nervous system infections: a review

**DOI:** 10.1007/s10072-025-08279-4

**Published:** 2025-06-02

**Authors:** Junan Hu, Wei Yu, Jiating Cui, Lun Zhang, Wangfang Yu

**Affiliations:** 1 Department of Neurosurgery, Beilun District People’s Hospital, Ningbo, 315800 Zhejiang China; 2 Department of Radiology, Beilun District People’s Hospital, Ningbo, 315800 Zhejiang China

**Keywords:** PCNSIs, Antimicrobial resistance, Molecular diagnostics, mNGS, CRISPR-Cas systems, Artificial intelligence

## Abstract

Postoperative central nervous system infections (PCNSIs), including meningitis, cerebral abscesses, and implant-associated infections, represent critical complications following neurosurgical procedures. These infections pose significant risks to patient outcomes due to delayed diagnosis, escalating antimicrobial resistance, and limited therapeutic efficacy. Conventional diagnostic approaches, such as cerebrospinal fluid (CSF) analysis, microbial cultures, and neuroimaging, exhibit notable limitations in sensitivity, specificity, and rapidity. This review highlights transformative technologies reshaping PCNSI diagnostics, including molecular assays (e.g., quantitative PCR, digital droplet PCR), metagenomic next-generation sequencing (mNGS), CRISPR-based pathogen detection platforms, metabolomics, and advanced molecular imaging modalities. Furthermore, we address translational challenges in clinical adoption, including cost barriers, standardization gaps, and the need for interdisciplinary collaboration. Emerging artificial intelligence (AI)-driven strategies are proposed to optimize pathogen identification, predict antimicrobial resistance profiles, and tailor personalized therapeutic regimens.

## Introduction

Postoperative central nervous system infections (PCNSIs) represent a range of infectious diseases caused by the invasion of bacteria, viruses, fungi, or other pathogenic microorganisms into the intracranial space following neurosurgical interventions. These conditions include meningitis, brain abscesses, subdural or epidural abscesses, and craniotomy flap infections [1]. Studies indicate that PCNSIs are a prevalent complication in neurosurgery, accounting for 0.8–7% of intracranial infections [2]. Neurosurgical interventions, often involving craniotomy, extensive brain tissue exposure, or the use of implanted devices, inherently increase the risk of microbial infiltration. Once established, these infections significantly prolong hospital stays, escalate healthcare costs, and may result in severe neurological deficits or mortality[3]. In the context of modern medicine’s emphasis on precision and personalized care, reducing surgery-related infection risks and enhancing diagnostic and therapeutic efficiency are pivotal to improving patient outcomes and elevating the quality of neurosurgical practice [4].

However, the diagnosis and management of PCNSIs in current clinical practice remain fraught with challenges. Conventional diagnostic approaches primarily rely on clinical symptoms, routine blood tests, cerebrospinal fluid (CSF) analysis, microbiological cultures, and imaging assessments. These methods frequently fall short in precision, particularly when confronted with diverse pathogens, lengthy culture times, or organisms that are difficult to cultivate, leading to missed diagnoses or delayed treatment [5, 6]. Moreover, the complexity of neurosurgical procedures and interpatient variability further elevate postoperative infection rates [7]. Consequently, early detection and accurate identification of PCNSIs have emerged as critical research priorities. A range of innovative technologies—including molecular assays, high-throughput sequencing, mass spectrometry, and molecular imaging—have recently been developed [8–10], offering novel strategies and tools to enhance diagnostic efficiency and personalize treatment.

This review aims to synthesize the epidemiology, primary risk factors, traditional diagnostic methods, and their limitations in the context of PCNSIs. It systematically evaluates the emerging diagnostic technologies introduced in recent years, elucidating their clinical utility while addressing the challenges and potential solutions for their widespread adoption. By doing so, it seeks to provide a valuable reference for clinicians and researchers alike.

## Epidemiology

### Incidence and risk factors

PCNSIs are among the most frequent complications following neurosurgical procedures, with incidence rates varying depending on surgery type, patient demographics, and healthcare conditions. According to Pan et al. [2], the incidence of PCNSIs ranges from 0.8% to 7%. Complex neurosurgical interventions, such as craniotomy for tumor resection, intracranial aneurysm repair, and spinal surgery, are associated with elevated infection rates, often exceeding 5% [11]. While advancements in medical technology and strengthened infection control measures have led to a reduction in the overall incidence of PCNSIs in recent years, marked regional disparities persist [12]. In developed countries, high-quality medical infrastructure and stringent aseptic protocols correlate with lower infection rates. Conversely, developing nations experience higher rates, attributable to limited resources and inconsistent infection control practices [13].

**Fig. 1:**
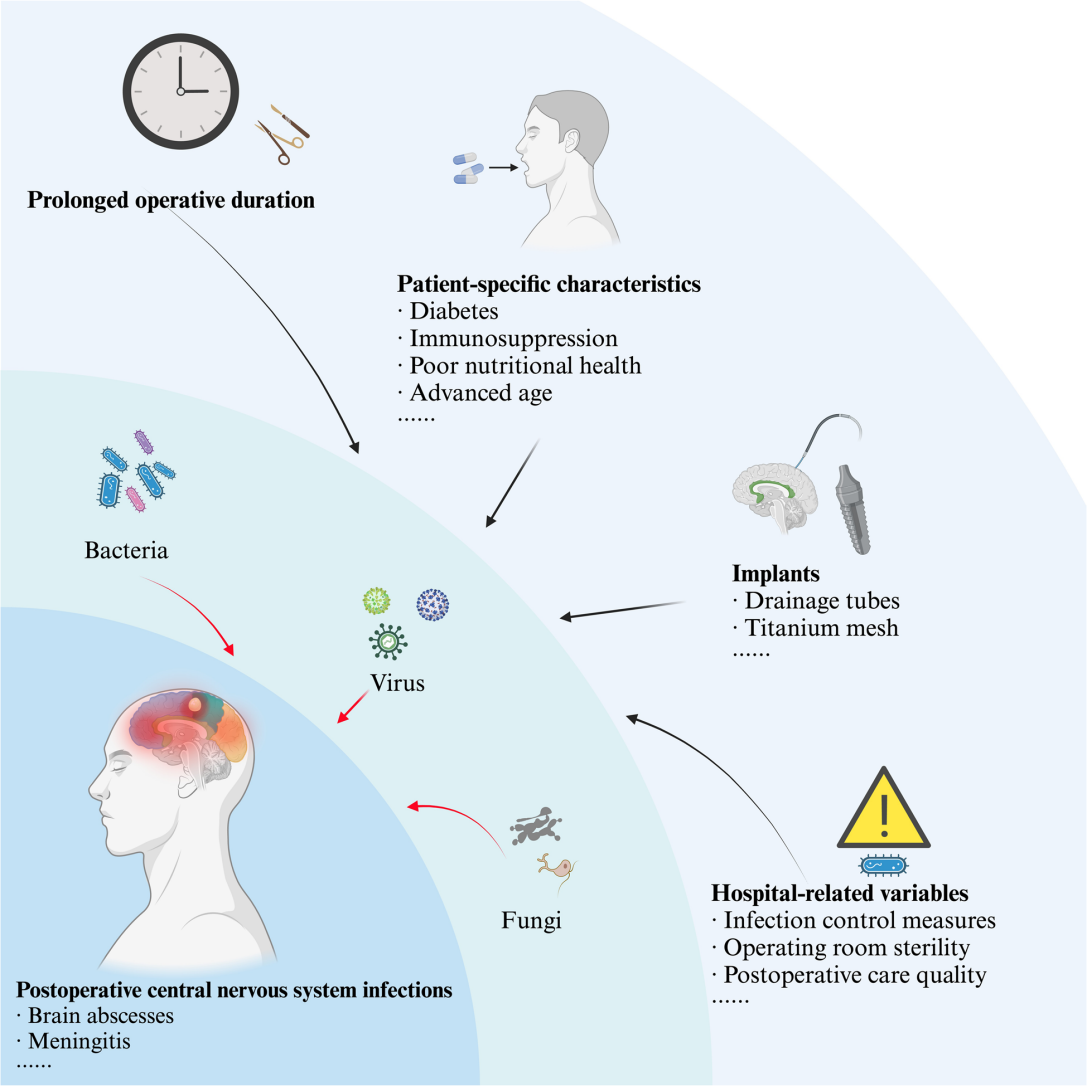
Factors contributing to postoperative central nervous system infections. This figure summarizes the key risk factors and causes of postoperative central nervous system infections, highlighting the roles of prolonged operative duration, patient-specific characteristics, implants, and hospital-related variables in facilitating pathogen spread

The development of PCNSIs is influenced by a combination of factors. Prolonged operative duration [14], repeated craniotomies, and the use of implants such as drainage tubes or titanium mesh significantly increase infection risk [15]. Patient-specific characteristics also play a critical role. Compromised immune status, as seen in conditions like diabetes or immunosuppression[16, 17], poor nutritional health, advanced age—particularly in elderly patients[18], and comorbidities such as chronic kidney or liver disease elevate susceptibility. Additionally, hospital-related variables, including the efficacy of infection control measures, operating room sterility, and postoperative care quality, directly affect infection rates [16]. These multifaceted risk factors underscore the complexity of preventing and managing PCNSIs across diverse clinical settings. Figure 1 provides a comprehensive overview of the interplay of these factors and their contributions to infection development.

### Pathogen distribution and evolution of antimicrobial resistance

The overuse of broad-spectrum antibiotics and the rise in invasive procedures have intensified antimicrobial resistance among pathogens causing PCNSIs, fundamentally altering their epidemiological profile [19, 20]. Gram-positive cocci, notably coagulase-negative staphylococci (CoNS) and Staphylococcus aureus, predominate in PCNSIs, with many strains exhibiting methicillin resistance[19]. This resistance complicates treatment, as conventional antibiotics often prove ineffective.

Additionally, Gram-negative pathogens such as Acinetobacter baumannii and Escherichia coli play a prominent role in certain regions, frequently displaying multidrug resistance (MDR) and further limiting therapeutic options[21, 22]. A. baumannii, in particular, accounts for a substantial proportion of infections in some hospitals, demonstrating high resistance to multiple antibiotics [19]. Concurrently, fungal infections—especially Candida and Aspergillus species—pose growing challenges, with increasing resistance to antifungals like fluconazole[23, 24].

The rise of resistant strains, including methicillin-resistant S. aureus (MRSA) and carbapenem-resistant Enterobacteriaceae (CRE), has spurred the development of novel antibiotic combinations, such as ceftazidime/avibactam, which show promise against MDR Gram-negative bacteria[25]. Nevertheless, resistance remains a formidable barrier to effective treatment. Advances in antibiotic development and precise resistance detection technologies, such as molecular diagnostics, are critical to addressing this issue[26, 27].

The evolution of resistance is driven by complex mechanisms. Horizontal gene transfer via plasmids accelerates the dissemination of resistance genes (e.g., blaKPC, mecA) among pathogens[28, 29]. Biofilm formation on implant surfaces physically impedes antibiotic penetration and enhances resistance, such as to vancomycin [30]. Furthermore, host immunosuppression—induced by postoperative glucocorticoid use or comorbidities like diabetes—weakens pathogen clearance, fostering resistant strain colonization[31].

The dynamic evolution of pathogen resistance imposes heightened demands on clinical anti-infective strategies. The limitations of traditional susceptibility testing (e.g., prolonged turnaround times, high false-negative rates) underscore the importance of molecular diagnostic tools [32]. Real-time monitoring of resistance genes (e.g., mecA, blaKPC) combined with rapid susceptibility profiling enables tailored therapeutic regimens, improving patient outcomes and curbing the spread of resistant pathogens[33, 34].

## Traditional diagnostic methods and their limitations

### Nonspecific early symptoms and diagnostic challenges

The early clinical presentation of PCNSIs often overlaps with noninfectious postoperative inflammation, such as surgical stress responses, hematoma resolution, or aseptic meningitis, compromising diagnostic sensitivity and delaying treatment [35]. Fever, a common occurrence in neurosurgical patients, frequently manifests shortly after surgery [36], yet widespread use of antipyretics like acetaminophen obscures the distinction between infectious and noninfectious etiologies [37]. Nonspecific symptoms—headache, altered consciousness, or mild neurological deficits—are prevalent in the early postoperative period but lack reliability as infection markers [38]. Classic meningeal signs, such as neck stiffness or Kernig’s sign, exhibit low positivity rates in bacterial meningitis and often emerge late, lagging behind microbiological evidence[39]. This symptomatic ambiguity complicates timely and accurate diagnosis, underscoring the need for more definitive tools.

### Limitations of laboratory diagnostics

Laboratory diagnosis of PCNSIs relies heavily on cerebrospinal fluid (CSF) analysis and inflammatory biomarkers, yet these methods exhibit significant limitations in sensitivity and specificity, particularly in early infection or complex clinical scenarios, increasing the risk of misdiagnosis. CSF analysis, a cornerstone of traditional diagnostics, faces multiple challenges that undermine its efficacy. Variability in cytological and biochemical markers, influenced by factors such as blood contamination in CSF samples, can obscure ventriculitis detection, as conventional methods depend on parameters like cell counts, lactate levels, and total protein [40, 41]. Elevated CSF leukocytes typically signal bacterial meningitis, yet in immunocompromised patients, sensitivity diminishes, elevating false-negative rates [42]. For instance, in HIV-associated cryptococcal meningitis, CSF leukocyte counts often fall below diagnostic thresholds due to impaired immune responses, hindering accurate detection[43].

Biochemical markers, such as the CSF glucose-to-blood glucose ratio, are widely used to differentiate bacterial meningitis from other forms. In bacterial meningitis, CSF glucose levels typically decrease markedly, yielding high specificity for bacterial etiology[44]. However, in fungal infections like candidal meningitis or tuberculous meningitis, this ratio may remain normal [45]. In tuberculous meningitis, while CSF glucose may decline, the reduction is less pronounced than in bacterial cases, rendering this marker insufficient for standalone diagnosis [46].

Inflammatory biomarkers, including C-reactive protein (CRP), procalcitonin (PCT), and interleukin-6 (IL-6), are routinely employed for infection screening. However, their interpretation requires caution. CRP, a widely used indicator, rises in early postoperative infection but also increases during noninfectious postoperative inflammation, limiting its ability to distinguish between these states [47]. PCT offers greater specificity and sensitivity for bacterial infections, with studies demonstrating significant elevations in PCNSIs while remaining stable in viral or noninfectious contexts [48]. Nonetheless, PCT levels can be confounded by surgical trauma or systemic inflammatory response syndrome (SIRS) [49]. IL-6, a key proinflammatory cytokine, elevates in both infection and inflammation, providing early diagnostic utility but lacking specificity unless combined with other indicators [50, 51]. These limitations highlight the need for integrative approaches to enhance diagnostic precision.

### Challenges in imaging-based diagnosis

Imaging modalities are indispensable for diagnosing PCNSIs, yet their sensitivity and specificity are constrained by technical limitations and lesion characteristics[52]. Computed tomography (CT) is widely utilized for its ability to rapidly identify acute complications like hemorrhage or cerebral edema. However, its sensitivity to early infection is limited, and postoperative artifacts or noninfectious inflammation can lead to misinterpretation. Magnetic resonance imaging (MRI), leveraging multi-sequence protocols such as T2-weighted and diffusion-weighted imaging (DWI), offers improved diagnostic capability [53]. Despite these advances, MRI specificity remains challenged. T2-weighted sequences often yield high false-positive rates due to meningeal enhancement, while DWI hyperintensity overlaps between abscesses and necrotic lesions complicates differentiation.

Research underscores MRI’s superior sensitivity, specificity, and diagnostic consistency compared to CT, positioning it as a valuable tool for confirming PCNSIs post-neurosurgery [53]. Additionally, MRI excels in detecting infection-related cerebrovascular complications, distinguishing them from noninfectious vascular pathologies—a critical factor for timely intervention[54]. Nevertheless, the spatiotemporal resolution of imaging techniques falls short in early-stage detection and precise differentiation, necessitating complementary diagnostic strategies.

## Breakthrough advances in emerging diagnostic technologies

**Fig. 2:**
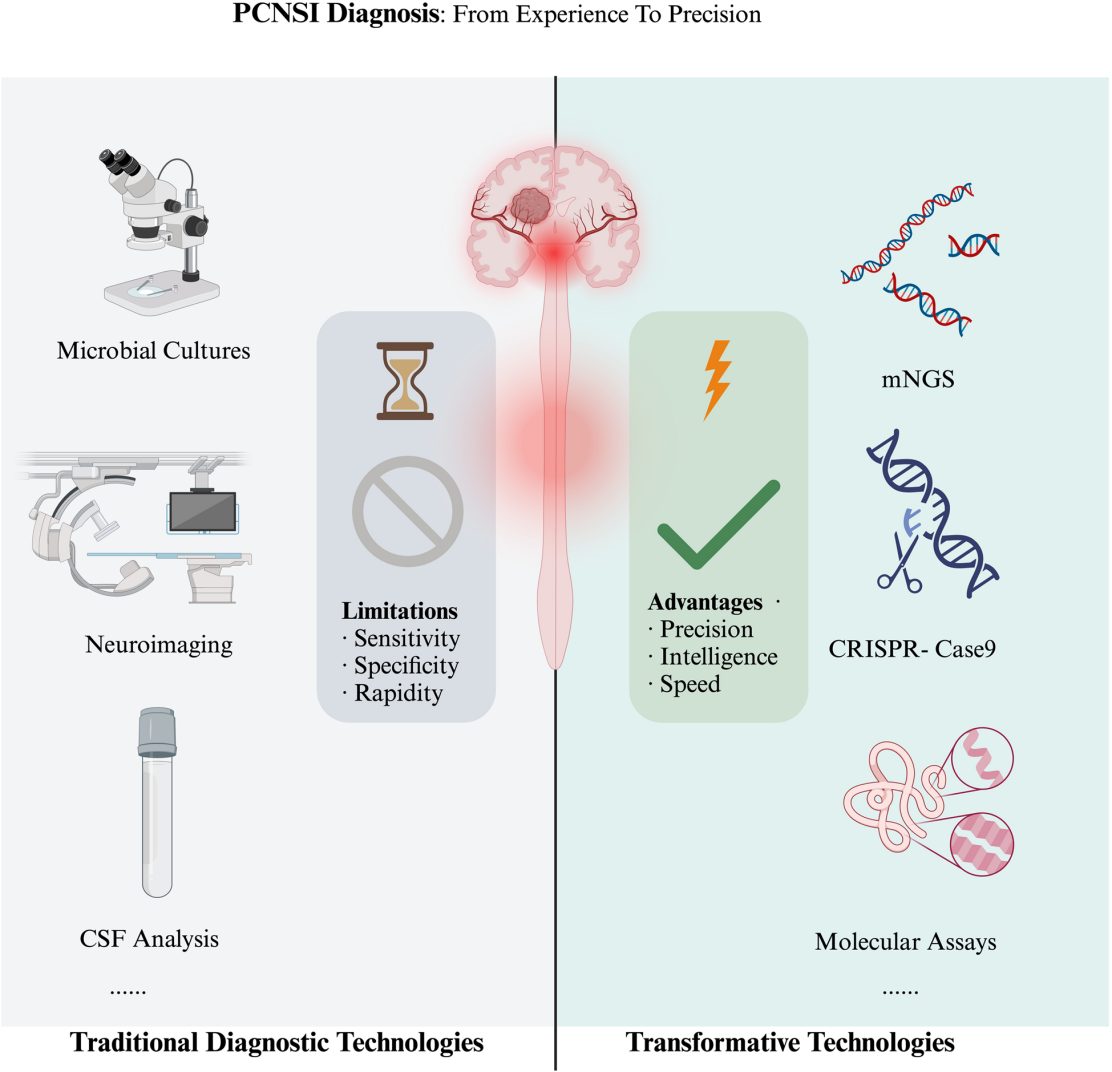
Evolution of PCNSI diagnostic technologies: from traditional to transformative. This figure contrasts traditional diagnostic techniques (e.g., microbial cultures, neuroimaging, CSF analysis) with transformative technologies (e.g., mNGS, CRISPR-Case9), highlighting the limitations of the former in sensitivity, specificity, and speed, and the advantages of the latter in precision, intelligence, and rapidity

Recent years have seen remarkable progress in diagnostic technologies for PCNSIs, transitioning from empirical evaluations to precision-based methods grounded in molecular biology, genomics, and advanced imaging[55–57].This section provides a systematic overview of key emerging technologies, exploring their underlying principles, advantages, and clinical applications. To highlight the evolution from conventional diagnostic approaches to these innovative techniques, Fig. 2 offers a comparative analysis of their respective strengths and limitations.

### Molecular biology-based detection

Polymerase chain reaction (PCR) technologies have evolved significantly beyond traditional nucleic acid amplification, with derivatives such as real-time quantitative PCR (qPCR), multiplex PCR, and digital PCR (dPCR) enhancing speed and accuracy [58]. qPCR offers distinct advantages in pathogen detection, particularly for early diagnosis of bacterial meningitis, fungal, or viral infections. Unlike culture-based methods, which require 24–48 h, qPCR delivers quantitative nucleic acid analysis within hours, markedly reducing diagnostic timelines [59]. Multiplex PCR enables simultaneous detection of multiple pathogen nucleic acids in a single reaction, proving invaluable for atypical presentations or culture-negative cases. Platforms like FilmArray exemplify this, screening bacteria, viruses, and fungi concurrently with high sensitivity and specificity, thus supporting rapid identification of mixed infections[60, 61].

Digital PCR (dPCR) further elevates diagnostic precision by partitioning reactions into thousands of microdroplets, enabling absolute quantification of target nucleic acids. Its heightened sensitivity detects low-concentration pathogen DNA, critical for conditions like tuberculous meningitis, where conventional methods often miss low-burden Mycobacterium tuberculosis due to insufficient sensitivity [62–64]. Moreover, dPCR’s quantitative precision provides pathogen load insights, aiding disease severity assessment and treatment planning[65].

### Metagenomic Next-Generation Sequencing (mNGS)

Metagenomic next-generation sequencing (mNGS) leverages high-throughput sequencing to indiscriminately analyze all microbial genetic material in a sample, overcoming the limitations of traditional culture methods. This technology excels in PCNSI diagnostics, offering rapid and accurate pathogen identification, particularly in complex cases [66]. A study that analyzed the CSF of 428 patients with CNS infections reported an mNGS detection rate of 34.6%, compared to 5.4% for conventional methods[67]. Its unbiased approach also identifies rare or novel pathogens, proving essential for cases eluding traditional diagnostics [68]. For example, Wang et al. reported a case of *Mycoplasma hominis* meningoencephalitis in a postpartum female, diagnosed using mNGS of CSF[69]. Beyond detection, mNGS elucidates pathogen genomic profiles, enhancing therapeutic decision-making, as demonstrated in community-acquired CNS infections where it uncovered pathogens missed by standard assays [70].

In acute settings, mNGS’s speed and comprehensiveness shine, with studies in emergency departments highlighting its utility for etiologically ambiguous cases [71].Third-generation sequencing platforms, such as Oxford Nanopore Technologies (ONT) and PacBio, further refine this approach with long-read capabilities and real-time output, improving resolution of genomic repeats and structural variants for precise pathogen identification and resistance profiling[72, 73].

### Metabolomics and mass spectrometry

Metabolomics, paired with mass spectrometry, has emerged as a powerful tool for diagnosing CNS infections by profiling metabolites to uncover disease-specific biomarkers and therapeutic targets. Liquid chromatography-mass spectrometry (LC–MS) is widely applied to CSF and serum, revealing metabolic shifts linked to neuroinflammation and infection[74, 75]. For instance, targeted metabolomics in COVID-19-related CNS infections identified 32 differentially abundant CSF metabolites, suggesting diagnostic potential[76]. In tick-borne encephalitis, metabolomics pinpointed tryptophan metabolism and the citric acid cycle as key pathways distinguishing infected from noninfected samples, offering novel biomarkers for differential diagnosis [77].

Additionally, CSF metabolomics detects neuroinflammation-associated metabolites, such as quinic acid and indoleamine 2,3-dioxygenase (IDO) pathway products, enhancing infection diagnosis and monitoring [78, 79]. Advances in mass spectrometry imaging (MSI) enable spatial mapping of metabolites in brain tissue, deepening insights into infection pathophysiology and supporting biomarker discovery [80]. These technologies not only facilitate early detection but also pave the way for personalized treatment strategies.

### CRISPR-cas systems

Originally identified as a bacterial adaptive immune mechanism against phages and plasmids, the CRISPR-Cas system has evolved into a versatile tool for PCNSI diagnostics and therapy [81]. By targeting specific pathogen genomes, CRISPR-Cas offers precise pathogen clearance, addressing the limitations of conventional treatments against multidrug-resistant bacteria [20, 82]. In CNS infections, where neurotropic viruses breach immune-privileged barriers, CRISPR-Cas’s gene-editing precision provides a novel therapeutic avenue [82]. Recent innovations in delivery systems enhance its in vivo stability and specificity, minimizing off-target effects and boosting clinical feasibility [83]. For example, Li et al. developed HOLMES, a CRISPR-Cas12a-based diagnostic platform, which detected Japanese encephalitis virus (JEV)[84]. These developments position CRISPR-Cas as a promising adjunct for managing PCNSIs, particularly in resistant or complex cases.

### Molecular imaging

Molecular imaging enhances PCNSI diagnosis and management by targeting pathogen metabolic signatures with specific tracers, enabling precise lesion localization and activity assessment. Combining magnetic resonance imaging (MRI) with positron emission tomography (PET) integrates anatomical and functional data, improving evaluation of infection extent and severity [85]. Nanotechnology has made significant advancements in medical imaging, particularly in multimodal PET and MRI, where nanoparticles serve as contrast agents to enhance imaging sensitivity and specificity[86]. Nanoparticle-mediated imaging further refines cellular process visualization, distinguishing pathological from normal tissue [87].

While 18F-FDG PET, based on glucose metabolism, aids in differentiating abscesses from tumors, its specificity is hampered by false positives from inflammation[88]. More specific tracers, like 68 Ga-citrate, exploit iron uptake pathways, achieving detection limits as low as 10^3^ CFU/g in preclinical models and showing promise in high-grade glioma imaging[89]. Similarly, 11C-methionine leverages metabolic differences to distinguish infection from gliosis, capitalizing on pathogen-host metabolic disparities [90]. With the widespread adoption of deep learning in medicine, AI models have demonstrated exceptional accuracy and efficiency in various neuroimaging tasks and disease-specific applications, significantly improving the diagnosis of intracranial infections [91].

## Challenges and future directions

### Clinical implementation challenges at the macro level

Despite the remarkable advantages of emerging technologies in diagnosing and treating PCNSIs, their widespread adoption faces significant hurdles. Foremost among these are cost and resource constraints: the high expense of metagenomic next-generation sequencing (mNGS) and molecular imaging systems (e.g., PET/MRI) imposes a substantial financial burden on resource-limited hospitals. For instance, mNGS per-test costs typically range from $500 to $3,000, depending on sample complexity and analysis depth[92]***.***

Additionally, gaps in standardization and professional training hinder progress. For instance, no unified guidelines exist for interpreting CRISPR-based diagnostics, while mNGS bioinformatics analysis demands specialized expertise, limiting scalability across healthcare tiers[93, 94]. Moreover, these technologies remain in early clinical validation stages, lacking robust evidence from large-scale prospective studies to inform guideline updates and clinical practice[95].

Strategies to mitigate these barriers include cost-optimization approaches. or equipment costs, lifecycle cost analysis optimizes procurement and utilization of high-cost imaging systems [96], while regional equipment-sharing hubs, such as multi-hospital consortia leasing PET/MRI systems, reduce access barriers in resource-limited settings[97]***.***Outsourcing maintenance contracts has proven effective in reducing expenses in low-income settings [98]. Low-cost detection methods, such as affordable reagents for KPC enzyme testing, offer viable alternatives for smaller laboratories [99]. Furthermore, deep learning-based super-resolution imaging can enhance MRI resolution without additional hardware investment [100]. Systematic reviews emphasize that procurement decisions for expensive medical equipment should balance technical, financial, safety, and clinical considerations through multidisciplinary input, ensuring economic and scientific rigor [101].

### Key technical challenges and targeted solutions

Beyond macroeconomic barriers, several core scientific challenges persist in PCNSI diagnostics and treatment. The table below summarizes four prominent issues, alongside innovative strategies and recent advancements addressing them (Table 1).

**Table 1:** Innovative diagnostic strategies for PCNSI challenges

Scientific challenge	Innovative strategy	Latest progress
Insufficient sensitivity in low-biomass samples	Microfluidic chip pre-enrichment	tenfold increase in pathogen capture efficiency[102, 103]
Human nucleic acid interference	CRISPR-Cas9 host DNA degradation	> 99% host DNA removal rate [104, 105]
Phenotypic-genotypic resistance mismatch	Single-cell sequencing for heterogeneous resistant subclones	Identification of mecA-negative MRSA clones[106]
Blood–brain barrier (BBB) penetration assessment	High-throughput drug screening via organoid models	BBB-on-chip enables rapid drug screening [107]

Low-biomass samples, such as CSF, often yield inadequate sensitivity. Microfluidic pre-enrichment boosts pathogen concentration, enhancing downstream mNGS or PCR accuracy [102, 103]. Human nucleic acid interference, which squanders sequencing resources and increases background noise, is mitigated by CRISPR-Cas9’s targeted degradation of host DNA, enriching pathogen signals [104, 105]. Discrepancies between resistance phenotypes and genotypes—exemplified by mecA-negative MRSA—are addressed through single-cell sequencing, revealing cryptic resistant subclones to guide personalized therapy[106]. Finally, assessing drug penetration across the blood–brain barrier (BBB) is critical for therapeutic efficacy. Humanized BBB-on-chip models facilitate rapid in vitro screening, reducing treatment failures from poor CNS drug delivery [107]. These models are experimental, with ongoing clinical studies validating their predictive accuracy for PCNSI therapeutics.

### AI platforms and future outlook

Artificial intelligence (AI) is poised to revolutionize PCNSI management by addressing these challenges. Low-cost targeted panels, optimized via AI, reduce diagnostic expenses while improving sensitivity and specificity for pathogen identification [108]. Regional pathogen databases, built through interdisciplinary collaboration across epidemiology, microbiology, and informatics, enable AI systems to adapt to local epidemiological patterns, enhancing diagnostic precision [109]. AI-driven diagnostic platforms, leveraging machine and deep learning, extract actionable insights from vast medical datasets, streamlining decision-making and minimizing human error [110, 111]. In pathogen identification and antimicrobial susceptibility testing, AI surpasses traditional method limitations, drastically shortening result turnaround times [112].

**Fig. 3:**
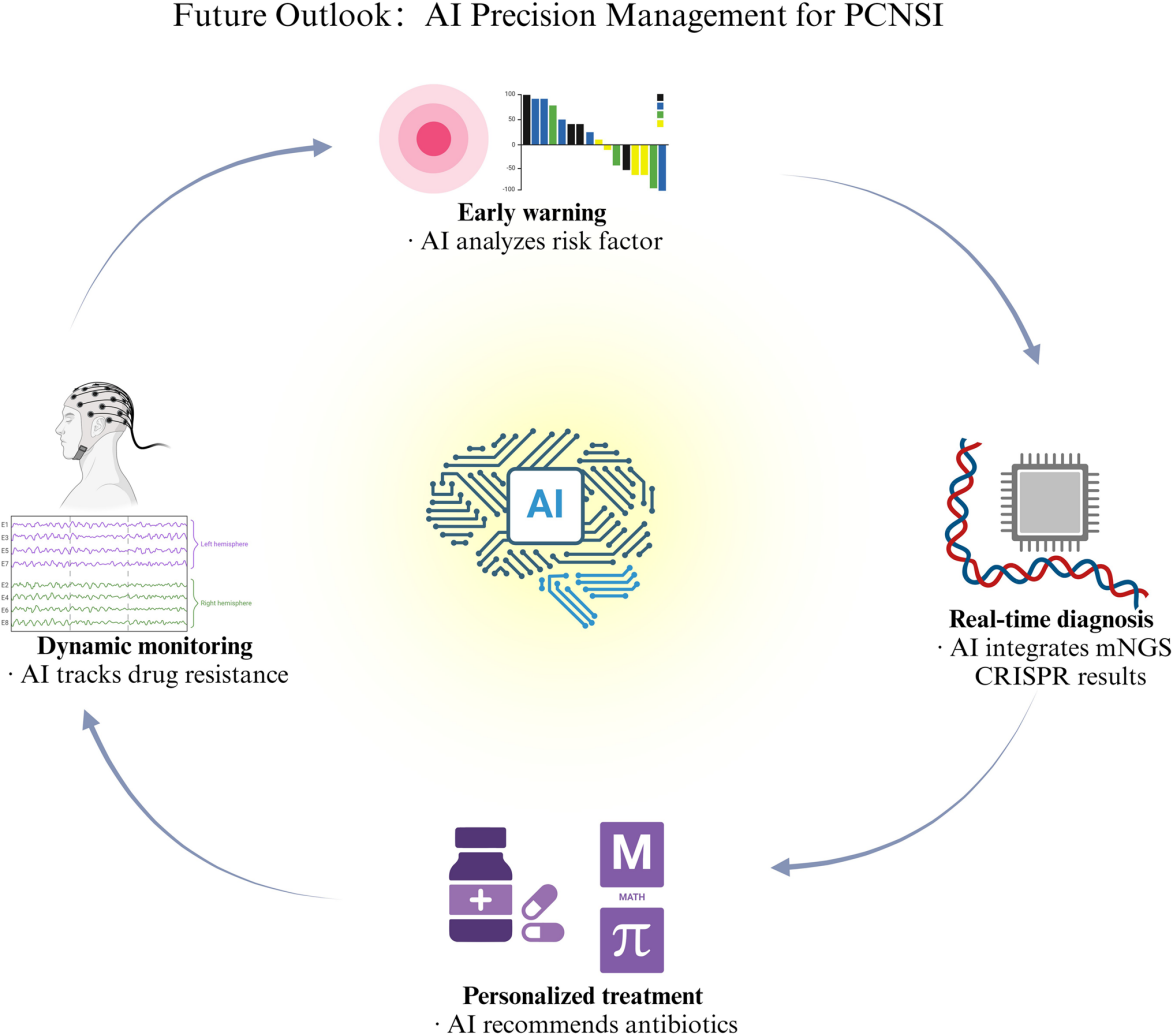
AI-driven precision management for postoperative central nervous system infections. This figure outlines the future role of AI in PCNSI management, including early risk factor analysis, dynamic monitoring of drug resistance, real-time integration of mNGS and CRISPR results, and personalized antibiotic recommendations

Looking ahead, integrating microfluidic enrichment, CRISPR-based host depletion, single-cell sequencing, and organoid models with AI platforms promises a comprehensive precision-management pipeline for PCNSIs—from rapid pathogen detection and resistance profiling to BBB penetration assessment and tailored treatment optimization. Figure 3 illustrates how AI can orchestrate these advanced technologies to create a cohesive framework for precision management, enhancing every stage of PCNSI care. Multidisciplinary collaboration and accumulating clinical evidence will drive these technologies toward transformative impact, positioning them as key enablers of precision medicine in neurosurgery [88].

## Conclusion

The diagnostic paradigm for postoperative central nervous system infections is transitioning from experience-based to data-driven frameworks, propelled by advanced technologies like metagenomic next-generation sequencing (mNGS), CRISPR-based detection, metabolomics, and molecular imaging. These innovations collectively enhance diagnostic sensitivity and accuracy, refining pathogen identification and fostering individualized treatment decisions [113]. Future progress hinges on developing low-cost targeted panels, regional pathogen databases, and AI-assisted diagnostics to broaden accessibility. Optimizing mNGS workflows and coupling them with AI-driven resistance prediction can further democratize infection diagnostics. With supportive policies and strengthened interdisciplinary collaboration, a closed-loop system of “precision early warning—real-time diagnosis—dynamic monitoring” is within reach, promising superior care for patients worldwide [114, 115].

## Data Availability

Data availability is not applicable to this article as no new data were generated or analyzed in this study.
